# Dupilumab as treatment of actinic prurigo suggests pathophysiologic mechanism of disease: A case series

**DOI:** 10.1016/j.jdcr.2024.01.039

**Published:** 2024-02-29

**Authors:** Catherine S. Barker, Lauren Sattele, Nicholas Strat, Sophia Daniel, Alan Snyder, Mark Siegel, Lara Wine Lee

**Affiliations:** a Department of Internal Medicine, Medical University of South Carolina, Charleston, South Carolina; b College of Medicine, University of South Carolina, Columbia, South Carolina; c College of Medicine, University of South Carolina, Greenville, South Carolina; d College of Medicine, University of Kentucky, Lexington, Kentucky; e Department of Dermatology and Dermatologic Surgery, Medical University of South Carolina, Charleston, South Carolina; f Department of Pediatrics, Medical University of South Carolina, Charleston, South Carolina

**Keywords:** actinic prurigo, dupilumab, IL-4, IL-13, pharmacology, photosensitivity, Th2

## Introduction

Actinic prurigo (AP) is a rare chronic photodermatosis typically seen in indigenous populations of North, Central, and South America.^1^ Approximately 93% of Latin American patients with AP are found to be positive for HLA-DR4, of which 80% have the DRB1∗0407 allele.^2^ These individuals are predisposed to type IV hypersensitivity reactions to UV radiation.^1^ Topical or systemic immunosuppression has been the standard of care. Here we present 2 patients with AP who significantly improved with dupilumab, an inhibitor of interleukins (IL) IL-4 and IL-13.

## Case report

### Patient 1

**Fig 1:**
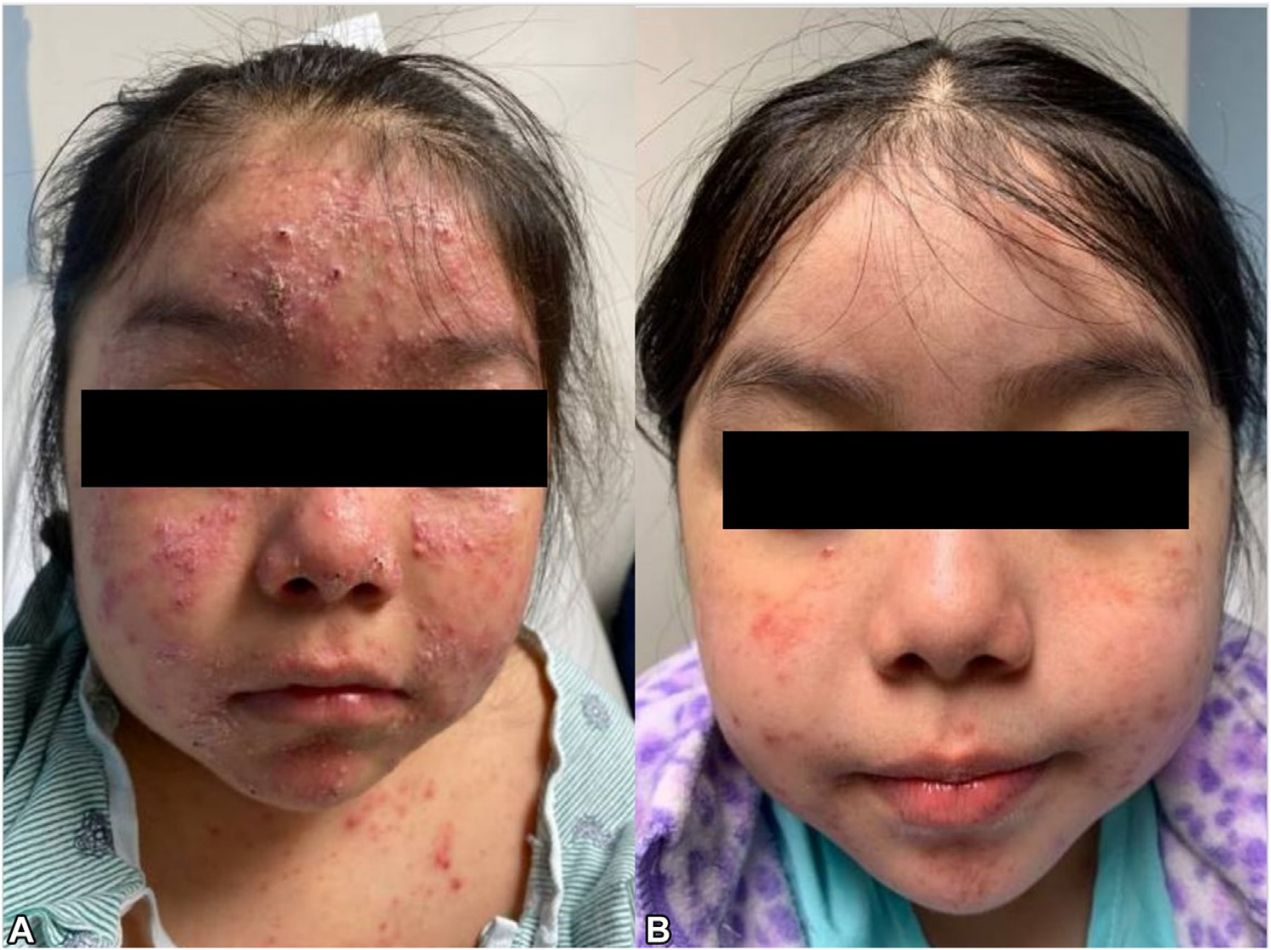
Actinic prurigo response to dupilumab, patient 1. **A,** Patient 1 presentation in clinic with a flare refractory to mometasone, tacrolimus, and photoprotection. **B,** Patient 1 following treatment with mometasone, photoprotection, and 2 months of dupilumab.

A 6-year-old female with known positive HLA-DR4 whose family was from Mexico had AP managed with sun protection and topical corticosteroids with continual residual disease and intermittent flaring. Following infection with influenza A, she experienced a persistent flare despite treatment with mometasone, tacrolimus, hydrocortisone, and strict sun avoidance (Fig 1, *A*). The patient endorsed severe pruritus and pain; she had a severe papular eruption on the face, trunk, and extremities. We prescribed a loading dose of dupilumab, continued mometasone 0.1%, and sun protection. Four weeks later, the patient reported significant improvement with decreased pruritus. Skin exam showed clearance on the back and abdomen with dramatic improvement of facial erythema and papules (Fig 1, *B*).

### Patient 2

**Fig 2:**
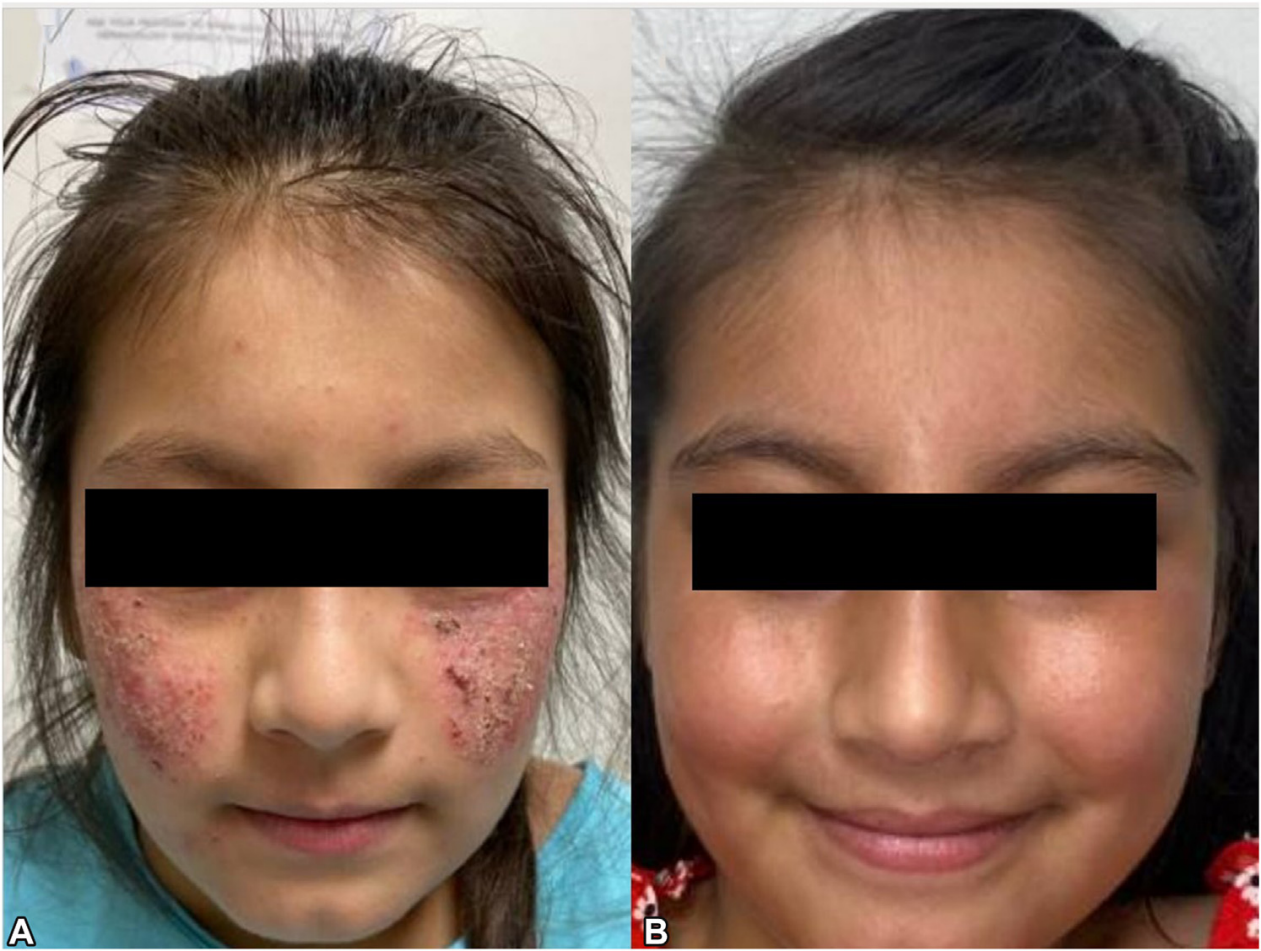
Actinic prurigo response to dupilumab, patient 2. **A,** Patient 2 on initial presentation in clinic. **B,** Patient 2 on triamcinolone and 1 month of treatment with dupilumab.

A 7-year-old female presented with a pruritic rash of the cheeks, arms, legs, and trunk triggered by heat and sun exposure. Her family was from Honduras. On exam, she had excoriated papules and erythematous plaques on the arms, legs, and cheeks (Fig 2, *A*). We suspected AP given her ethnicity and photodistribution of the rash. The patient had a good initial response to topical triamcinolone, mometasone, and sun protection measures but continued to flare intermittently. We initiated dupilumab. After 4 weeks, she reported significant improvement with no active rash (Fig 2, *B*).

## Discussion

Topical immunosuppression with corticosteroids and/or calcineurin inhibitors is appropriate for mild-to-moderate disease. For severe AP, thalidomide and cyclosporine are classically used treatments, but patients frequently flare upon withdrawal thus making chronic use necessary.^1^ AP patients usually require chronic therapy, and the potentially severe side effects particularly with chronic use of thalidomide and cyclosporine limit their utility, especially in the pediatric population.^1^^,^^3^ Antimalarials and phototherapy with narrowband UV-B or psoralen plus UV-A have also been used to desensitize patients to UV activity.^4^

**Fig 3:**
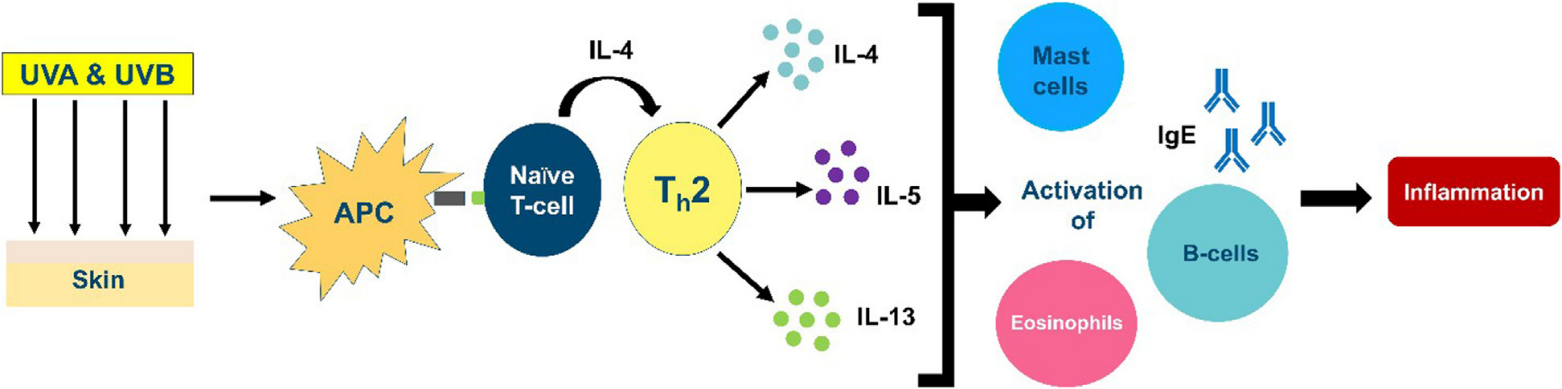
Proposed Th2 mediated mechanism of actinic prurigo. *APC,* Antigen presenting cell; *Ig,* immunoglobulin; *UV,* ultraviolet.

Current evidence suggests that AP is driven by a type IV hypersensitivity response to UV-A and UV-B radiation.^4^ AP may involve both Th1 and Th2 T-lymphocyte mediated response based on high levels of IgE, presence of eosinophils and mast cells, and tumor necrosis factor-⍺ activation upon exposure to UV-A.^1^^,^^4^ Th1 activity is postulated as the reason for response to thalidomide.^1^ Dupilumab treats chronic allergic inflammatory disorders, disrupting Th2 activation via inhibition of IL-4⍺ receptor, which modulates both IL-4 and IL-13 activity.^5^ AP, therefore, may be at least partially propagated by Th2 cells which produce IL-4, IL-5, and IL-13, driving production of B cells, IgE, and IgG4 (Fig 3).^1^ Dupilumab has resulted in sustained clearance of chronic AP in 2 previous case reports.^1^^,^^6^ Its reported effectiveness, relatively safe side effect profile, and minimal monitoring and administration may make dupilumab a drug of choice compared to other systemic therapeutics. Our patients had resolution of flares following a month of treatment with dupilumab.

Dupilumab is a novel option to treat AP. The pathophysiology behind this disease remains somewhat unclear although current evidence suggests that dupilumab’s inhibition of IL-4R⍺ may decrease the disease burden and is supported by the results of these cases. Future clinical trials are warranted to further elucidate the effectiveness of dupilumab in this unique photodermatosis.

## Conflicts of interest

Dr Wine Lee has received fees from Sanofi and Regeneron. Dr Barker, Author Sattele, Author Strat, Author Daniel, Dr Snyder, and Dr Siegel have no conflicts of interest to declare.
